# The Story of the Insane from Year to Year

**Published:** 1904-01-23

**Authors:** 


### THE STORY OF THE INSANE FROM YEAR TO YEAR.

Sixteenth Annual Series.

### CUMBERLAND AND WESTMORLAND ASYLUM.

Here on the first of January 664 were in residence, and at the end of the year the number had increased to 694. There were 142 first admissions, 48 readmissions, 70 discharged recovered, 16 discharged relieved, and 61 died. The average daily number resident was 677, and the total number under treatment was 844. Calculated on the admissions the recoveries were 36·8 per cent., and calculated on the average number resident the deaths were 9 per cent. Of the year's deaths 30 were caused by cerebral and spinal diseases, 4 by pneumonia, 4 by consumption, and 1 by enteritis. Various moral causes account for the insanity in 38 of the admissions; and among the physical causes, heredity is responsible for 61, previous attacks for 50, and intemperance in drink for 22. When speaking of the causes of insanity in the year's admissions, Dr. Farquharson says that hereditary influence was known to exist in 32 per cent., and that it probably existed in a much larger proportion. He adds that "the evils resulting from faulty marriages cannot be over-estimated. Public opinion has not as yet advanced sufficiently to enable the people to include hereditary predisposition in the list of preventible causes of insanity; but it is hopeless to expect any great diminution in the amount of such diseases till the laws of nature and heredity are much more widely appreciated and acted on." How true is all this, and yet how futile is the attempt to get in into the minds of the people; but Dr. Farquharson must not be discouraged, although he will find himself like one crying in the wilderness for many a year to come. The asylum is full; but little relief can be expected from transferring any of the patients from the asylum to the workhouses, or from discharging the private patients, many of whom would certainly find their way back as paupers, and so it has been decided to build extensions which are expected to cost £37,000. It cannot be said that this decision has been arrived at too soon—in fact, it is too late to save boarding-out at 14s., which will mean a loss of 4s. 8d. on each case to the local ratepayer. Last year the increase in the number was 30, which is something like 4 per cent. on the number resident. Garland's Asylum has often sought credit for the high percentage of recoveries, and now that more than a generation has passed over its head, it must be expected to have an equally high proportion of admissions, for recoveries in one generation must mean increase of insanity in the next, a reflection as painful as the statement is true. It would seem that 37 per cent. of the male staff and 48 per cent. of the female staff have not completed one year's service. The weekly charge for maintenance was 9s. 4d. per head.

### SURREY COUNTY ASYLUM AT BROOKWOOD.

On the first of January the asylum contained 1,002 patients, and at the end of December the number had risen to 1,070. There were 298 first admissions, 38 readmissions, 91 discharged recovered, 29 discharged relieved, and 93 died. The average number resident was 1,036, and the total number under treatment was 1,338. The recoveries stand at 29 per cent. of the admissions, and the deaths at 9 46 of the average number resident. Of the year's deaths, 43 were caused by cerebral and spinal diseases, 5 by pneumonia, and 8 by consumption. Mental anxiety, domestic trouble, and other moral causes were supposed to account for the insanity in 93 of the admissions, and among the physical causes, hereditary influence was responsible for 86, previous attacks for 36, and intemperance in drink for 48. The recovery rate is below the average of county asylums, but the material which Dr. Barton had to work on was most unpromising, and at the end of the year hardly 5 per cent. of the 1,070 patients then in residence could be deemed curable. Many were old and feeble, 127 were epileptic, 22 were general paralytics, and 104 were imbeciles or idiots. It is when examining statistics of this kind that we are sceptical concerning the high percentage of cures promised to us by asylum medical officers who clamour for acute hospitals in which to treat their recent cases. Classes for instruction of the attendants and nurses continue to be held with satisfactory results, but the duration of service of the nurses does not seem to lengthen at Brookwood, for we notice that 40 per cent. of them had less than one year's service. No mechanical restraint was used during the year, but several female patients were secluded. The clerk to the visitors received a pension of £100 a year, and the gardener one of £31. The committee express "their approval of the management of the asylum by Dr. Barton." The weekly maintenance rate was 12s.

